# In search of the perfect *conditions* for Suzuki–Miyaura cross-couplings

**DOI:** 10.3389/fchem.2026.1814565

**Published:** 2026-05-14

**Authors:** Bruce H. Lipshutz, Erfan Oftadeh, Marco Ortiz

**Affiliations:** Department of Chemistry and Biochemistry, University of California, Santa Barbara, CA, United States

**Keywords:** chemistry in water, new ligand, Pd and Ni catalysis, sustainability, Suzuki–Miyaura couplings

## Abstract

Developments in Suzuki–Miyaura (SM) couplings are discussed from a historical standpoint as they are especially valued C–C bond-forming reactions in the fine chemicals industry today. However, discussion from the increasingly important standpoint of sustainability is also considered when looking for conditions leading to the perfect coupling. Thus, several procedures now exist leading to the desired SM couplings, which reproducibly occur in or on water, rather than in traditional, petroleum-based organic solvents. Moreover, procedures based on low loadings of palladium catalysts are also in hand that ensure that this precious and, at one time, endangered metal will be available indefinitely. In addition, the beginning of a new series of ligands is discussed that, from a life cycle assessment perspective, meet the required criteria (including method of formation, avoidance of intellectual property (IP) issues, number of steps involved, cost, and effectiveness) and should, therefore, feature prominently in the development of SM couplings. This goal may even be more attainable considering the recently reported use of “on dirty water” conditions, which enables close-to-neat reactions to be combined with a highly concentrated “on water” effect.

## Introduction

**FIGURE 1:**
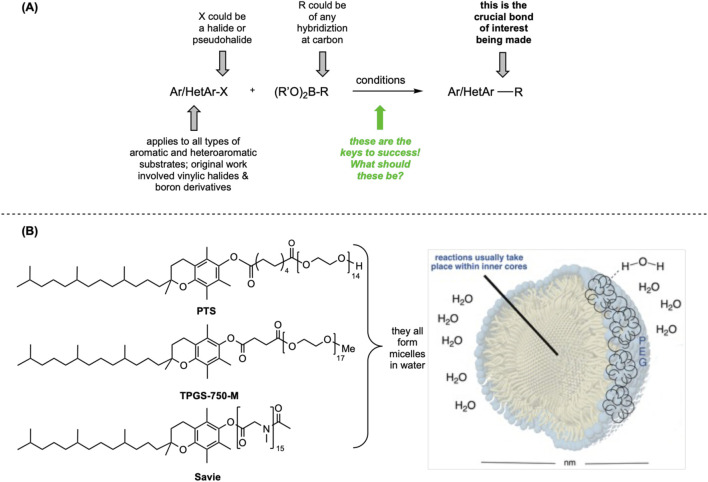
**(A)** General nature of Suzuki–Miyaura couplings. **(B)** “Designer” surfactants forming nanomicelles in water.

When the “Suzuki” coupling was first reported in 1979 (Miyaura et al., 1979), the field of chemistry was in a very different place. While society was continuing to increase its dependence on petroleum-derived products, chemistry (unknowingly?) became increasingly unsustainable, as it is to this day, in its use of organic solvents in which most reactions of organic chemistry are run. The *12 Principles of Green Chemistry (Anastas and Warner,* 2000*)* were decades away from making an appearance; the existential problems currently faced by the world—particularly climate change—were barely on the horizon. However, times have dramatically changed and so have organic chemistry and the associated cross-couplings that were then called Suzuki couplings. These changes go far beyond the names by which one today refers to these Nobel Prize-winning C–C bond-forming reactions, “Suzuki–Miyaura” (SM) couplings (Figures 1A,B).

What makes for a *perfect* Suzuki–Miyaura coupling *today*? What goals should we be striving to meet? Standards are very different from those in place when SM couplings were first conceived. Historically, while successful reactions focused mainly on yields, they are far from the only variable of consequence, not to mention that this process was initially done under homogeneous conditions. Nowadays, it is possible to form the same bonds using heterogeneous catalysis. Thus, when incorporating traditional synthetic parameters that are indicative of the value of SM couplings, and adding the timely concepts associated with the *12 Principles* (Anastas and Warner, 2000; Lipshutz and Handa, 2026), the list today might include several items leading to a perfect SM coupling (List 1):

List 1. Presumed items resulting in a perfect SM coupling:

1. Reaction efficiencies that appear to be independent of coupling partners
2. Water as the reaction medium, minimizing the use of organic solvents throughout the process
3. Minimizing investment of energy by running reactions preferably at or close to rt
4. Reducing the loadings of the Group 10 metal catalyst, whether Pd or an Earth-abundant metal
5. Increasing the observed rates of couplings
6. Eliminating residual metal from coupling products to meet FDA standards
7. Developing new ligands for catalysts, with preparations that rely on environmentally responsible syntheses

With more than 4 decades of “progress” in cross-coupling chemistry, but now with SM reactions in a very different world, where are we?

## Discussion

Research along these lines (*i.e.,* developing the perfect SM coupling) began for us in 2006 in this then-new area called “green chemistry.” We received help from several sources: (1) the ACS Green Chemistry Institute’s teachings, in which the “elephant in the room” was, and unfortunately still is, organic solvents (ACS Green Chemistry Institute, 2023); (2) the Olme review, attesting to a lack of Pd catalysis for reactions run under aqueous micellar catalysis conditions (Dwars et al., 2005) and lastly (3) Nature, with 4 billion years of chemistry in water to its credit. Certainly, reaction media have helped address the problem, such as the recommended use of “greener” organic solvents (Prat et al., 2016), deep eutectic solvents (Ferreira and Sarraguça, 2024), the use of fluorocarbons (Matsubara and Ryu, 2011), or even cold CO_2_, and others. However, given the role that water has played in the evolution of humanity, the answer seemed then, as it does today, almost too obvious. Nature’s choice has always been water (Warner, 2018).

Once it was realized by others, for example*,* Sharpless and his team, who knew that “solubility does not matter-…” (Bardi, 2005), that Nature had solved the solubility problem, in general, and in humans, given the seemingly endless list of essential biomolecules that are not water-soluble (*e.g*., coenzyme Q_10_, vitamin E, and many polypeptides), the question became, “what is Nature’s ‘secret’?” The answer is, and always has been, within us: water-insoluble compounds are localized in the membranes, vesicles, and micelles present in every human being. The question to be addressed, therefore, *en route* to a perfect SM coupling, was how to modernize a micelle so that a Group 10 metal-catalyzed reaction could be accomplished without recourse to the petroleum-based reaction media that have been used for decades (*i.e.*, organic solvents).

Early on, it was shown that SM couplings could be run in water using 2 wt% (Lipshutz and Ghorai, 2008) of a nonionic surfactant, “PTS” (PEGylated α-tocopheryl sebacate) (Lipshutz et al., 2008a; Lipshutz et al., 2008b) at rt (*i.e.,* 20 °C–45 °C). Good isolated yields of the desired biaryls were obtained, providing encouragement that this approach would be viable to the exclusion of organic solvents (Figure 1B) for several other types of couplings. The choice of vitamin E as the lipophilic interior of each nanomicelle was based, again, on evolution (Borowy-Borowski et al., 2000; Borowy-Borowski et al., 2010; Borowy-Borowski et al., 2013) although the key point is that modern organic reactions, such as SM couplings, do not require a medium containing pure organic solvents and usually require some water to be in the reaction mixture.

A far more efficient synthesis of PTS gave rise to the workhorse surfactant TPGS-750-M (*i.e.,* methyl PEGylated α-tocopheryl succinate; “TPGS”; Figure 1B) (Lipshutz et al., 2011), which utilizes a 4- rather than 10-carbon linker, and a methylated PEG terminus. The most recently reported “designer” surfactant is Savie (Kincaid et al., 2023) (Figure 1B), which is a non-PEGylated and biodegradable version of both PTS and TPGS. Fortunately, this alternative surfactant afforded the same or better outcomes from SM couplings. Once the nanomicellar medium was established as effective, comparison reactions between green conditions (*i.e.,* in aqueous micellar media) versus those using more traditional mixes of organic solvents with small percentages of water, suggested that ligands developed for organic synthesis made and used in organic solvents are not always the ideal choice for couplings run under aqueous micellar catalysis conditions; that is, the “rules” for carrying out name reactions, including SM couplings in water, are different (Lippincott et al., 2019; Lipshutz, 2018)!

Status check: Based on these advances, how close are we to a perfect coupling?

Answer: Items (1)–(3) on **List 1** appear to be within reach.

To reduce the amounts of “endangered” and costly palladium needed for SM couplings (Moghadam et al., 2020), the new “rules” associated with chemistry in water were exploited (Lipshutz, 2018; Lippincott et al., 2019), including increasing the ligands’ binding constant with the micellar non-polar interior, thereby slowing the exchange between the catalyst through the surrounding aqueous medium, which translates into the need for less catalyst. Moreover, because the interior of each nanomicelle offers a far more concentrated environment (*i.e.,* >2 M) relative to what is typically used in organic media (Myers, 2020), it came as no surprise that low loadings of Pd-containing catalysts are very effective at mediating these couplings (*vide infra*).

Once Nature’s secret for reducing the amount of metal catalyst for various reactions was discovered (interpreted as “new” rules that are, in fact, very, very old)—in this case, for Pd catalysis of SM couplings—another of the many benefits associated with chemistry in water was also uncovered: reduced levels of residual metal expected in the coupled products. In other words, by putting less Pd into an SM coupling, there is less residual Pd in the product. Aside from this intuitive observation, there is also an advantage that the lipophilic ligand complex, which binds to the metal, is designed to be highly insoluble in water; hence, the resulting catalyst has no incentive to leave the micelle and enter the water. Thus, while the product is certainly undergoing exchange and eventual precipitation from the water, perhaps not surprisingly, levels of residual Pd rarely exceed the FDA’s permitted daily exposure (PDE) of 10 ppm/day (U.S. Food and Drug Administration, 2022).

**FIGURE 2:**
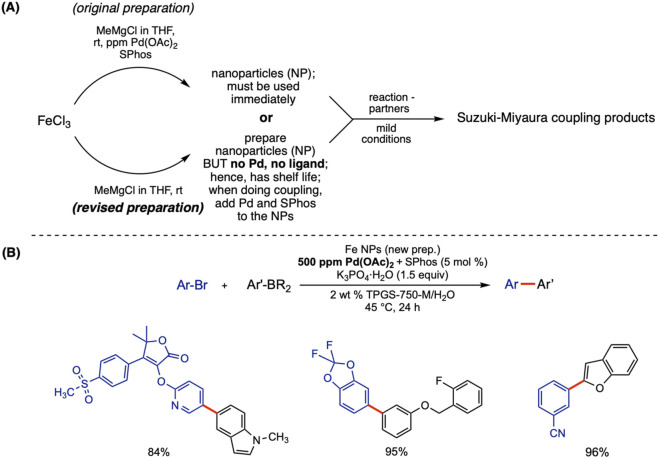
**(A)** Initial and modified routes to Pd-containing NP catalysts and **(B)** examples of Suzuki–Miyaura couplings run under aqueous micellar conditions using heterogeneous NP catalyst conditions.

So far, these environmentally responsible processes are run under homogeneous conditions, in which the catalysis occurs inside the micellar cores (see Figure 1B). However, the option to pursue *heterogeneous* catalysis is an attractive alternative mode, potentially with limited amounts of Pd, presumably located on the surface of the nanoparticles (NPs) created for this purpose. The same argument can be made given the many uses of metal-organic frameworks (MOFs) (Lawrence et al., 2022; Rabiei, 2026). Using very inexpensive FeCl_3_ as the starting material (Figure 2A), new NPs could be prepared, to which only small amounts of Pd (in the form of Pd(OAc)_2_) are initially added, along with the ligand (Handa et al., 2015b).

A far more intriguing aspect to this study, however, was recognition that the FeCl_3_ itself, obtained from sources that varied from vendor to vendor, contained varying levels of Pd salts as an “impurity.” Thus, because elemental iron is mined in different parts of the world prior to chlorination, it seemed obvious that each commercial source would have its own impurities, with palladium potentially among them (along with Ni, Cu, and other transition metals). ICP-MS determined that the FeCl_3_ from a supplier in India contained 320 ppm Pd. Because it formed NPs capable of catalyzing SM couplings when treated with MeMgCl in the presence of SPhos, the cost of the Pd used was … zero (Figure 2A).

Control reactions using highly purified FeCl_3_ + 320 ppm Pd(OAc)_2_ documented that the amount of Pd in the FeCl_3_ was the same amount needed to effect these couplings, although ultimately, 500 ppm (0.05 mol%) of the Pd catalyst was routinely used (Figure 2B). Although this commercial source of FeCl_3_ no longer contains this much palladium (it was immediately removed from the market after the article was published), the study nonetheless showed that (a) NPs could be fashioned containing very low loadings of Pd that effectively catalyze SM reactions in water; and (b) Nature provides “gifts” that are already in place, waiting to be discovered.

Status check: Are we almost there, that is, at a perfect coupling?

Answer: Yes; items 1–6 (**List 1**) seem to be under control, but #7 may be the biggest challenge.

It has taken >15 years to “learn” how to do cross-couplings in an aqueous micellar medium involving either homogeneous or heterogeneous catalysis, mainly based on Pd. However, while under development, the background “noise” continued to increase (rightfully) and kept getting louder. It’s content: A holistic approach is needed! A life cycle assessment (LCA) is the next frontier in green chemistry (Jessop and MacDonald, 2023). Identify the “hot” spots before starting to make anything (Jessop and MacDonald, 2023)! These ideas have been “in the works” for years and once here in full are not going away.

What this means in striving for a “perfect” coupling is that all components associated with the intended SM coupling must now be viewed in a different, more revealing light. In other words, the question is: What is the chemistry leading up to these ligated catalysts being used in SM couplings? Because the starting materials (*i.e.,* reaction partners) are identified on a case-by-case basis, that leaves only one parameter, in addition to the other three (*i.e.,* the solvent, reaction temperature, and time that determine energy invested, and low loadings of a metal catalyst), that is both extremely important and yet totally exposed: the *ligand*.

What can be done about this crucial variable? Most practitioners agree that the ligand can easily make the difference between success and failure, and that ligand screening is usually essential to arrive at the best conditions for the intended catalysis (Michaelis and Luxembourg, 1895). However, from a holistic perspective, ligands that enter the screening process come from somewhere, and, in general, whether a ligand is newly created or an established commercial item, it must be made. This only adds to the notion that more time and resources need to be invested, even prior to using catalysis for bond construction. On top of that comes the potential for addressing IP issues, should the selected ligand be used at scale for commercial purposes. Something is wrong with this picture: how would Nature deal with this need, having had so much time to solve this problem?

**FIGURE 3:**
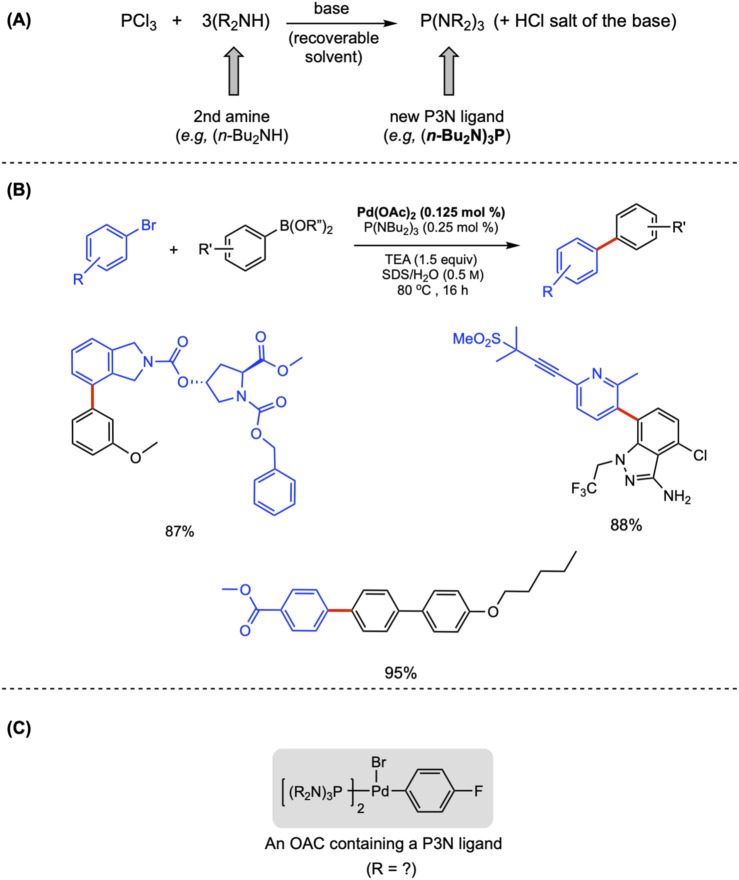
**(A)** Preparation of P3N ligands. **(B)** Examples of products using P3N-ligated Pd as a catalyst for SM cross-couplings. **(C)** An oxidative addition complex made with P3N ligands.

The pathway towards a solution to many problems that Nature has chosen over time is usually simple; take the same readily available atoms and combine them in a way that, in a single step, affords an arrangement leading to a general solution, in this case, for ligand design. Hence, using the same elements: C, H, N, and P, affords arrangements that place three nitrogen atoms on phosphorus, or “P3N” ligands (Oftadeh et al., 2025) derived simply from PCl_3_ and three equivalents of a secondary amine (Figure 3A). The initial species discovered, (*n*-Bu_2_N)_3_P, goes onto palladium in a 2:1 ratio to generate a catalyst that is very effective at mediating SM cross-couplings (Oftadeh et al., 2025). The two components that go into its 1-step formation at room temperature are quite inexpensive and, hence, all things considered, translate into essentially little to no cost to make, other than time (which is minimal). Use of (*n*-Bu_2_N)_3_P allows for ppm levels of precious metal (palladium) and late-stage, highly functionalized educts to smoothly participate, thus making these ligands very competitive on all fronts (Oftadeh et al., 2025).

As effective as (*n*-Bu_2_N)_3_P may be (see the examples as illustrated in Figure 3B), improvements are anticipated given the availability of secondary amines, as well as future options for preparing their on-cycle oxidative addition complexes (OACs; Figure 3C; Iyer et al., 2024), which should help increase reaction rates, seemingly independent of the nature of the groups on the aryl or heteroaryl halide (as observed from the examples in Figure 3B).

Is there an alternative to hetero- and homogeneous catalysis involving aqueous micellar media?

**FIGURE 4:**
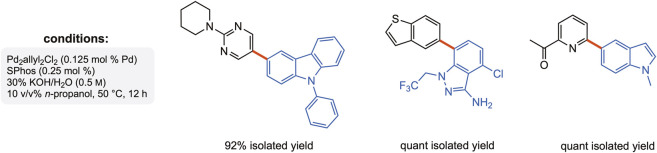
SM couplings using 30% KOH in water (between aryl bromides and boronic acids).

Yes, but again, one must look to Nature, which suggests a high probability that this option has been “missed.” It is in the category of Sheldon’s “The best solvent is no solvent … ” (Breslow, 1991; Kitanosono et al., 2018; Narayan et al., 2005; Sheldon, 2007), and yet, it is not in the “neat” reaction category. While the “on water” effect has been known for decades (Ortiz et al., 2026), it relies on pristine (*i.e.,* pure) water, which is far from what Nature has used over the millennia. In other words, chemistry has been done “in dirty water” from the beginning of time; so, what was “in the water”? Suffice it to say that by using KOH, and lots of it (and hence, a viscous, high pH that also serves as a base for several Pd-catalyzed reactions), it is now possible to use this phenomenon reproducibly and productively for SM couplings (Figure 4) with (a) functionalized reaction partners; (b) base-sensitive groups present, such as esters and nitriles; (c) easy recycling of the dirty water; and (d) low loadings of Pd.

However, the BIG question remains:

Are Earth-abundant metals as catalysts competitive for SM couplings?

Although all the key elements now seem to be well understood for eventually realizing the “perfect” SM coupling conditions, there remains one hurdle that has not been met … at least not yet. Current arguments for switching from Pd- to Ni-based catalysis, or any other Earth-abundant metal (EAM), today are virtually all hype with little to no substance, given that the catalysts are used for reactions completed in organic solvents (Lipshutz, 2026). However, there is also no debate that IF a Ni-catalyzed coupling existed that could catalyze under *aqueous* conditions and which requires the same (low) loadings of Ni (or any EAM) now possible with Pd …, well, that would be very close to “perfection.” Put another way, the future is likely to be in Ni catalysis simply for reasons of cost and availability, but only if the reactions can run without being environmentally egregious.

The idea that SM couplings can be catalyzed using Ni in Nature’s preferred medium, water, is not new, having first appeared more than a decade ago (Handa et al., 2015a). While rarely cited by those currently using Ni in organic solvents, the reality is that the amount of Ni used in that study (2 mol%) is excessive because, at this loading, too much metal is likely to be present in the product, thereby necessitating its removal. While there are claims that removing Ni is easier than removing Pd (Fantoni et al., 2025) (using equal amounts of this precious metal, which is no longer needed; *vide supra*), it still takes time, resources, and likely additional organic solvents, thus adding cost and, more importantly, contributing to climate change. Ideally, therefore, the perfect Suzuki–Miyaura cross-coupling will use ppm levels of Ni catalysis involving a reactive OAC, in addition to the other items on List 1, above.

**FIGURE 5:**
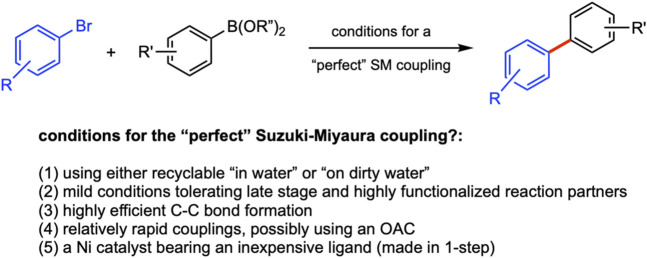
Proposed requirements for a “perfect” Suzuki–Miyaura cross-coupling.

Put another way, still to be discovered is a Ni catalyst that not only contains a new ligand with all the virtues found for new P3N ligands (*vide supra*), but that also can be used under aqueous conditions at low loadings such that the products contain levels of residual Ni below that allowed by the FDA (20 ppm/day) (U.S. Food and Drug Administration, 2022). We are on it (Figure 5).

## Conclusion

Given the huge role that Suzuki–Miyaura reactions continue to play in all areas of organic synthesis, the “perfect” cross-coupling that incorporates both traditional concerns (such as yields) and timely and important green aspects (as in environmental issues, including but not limited to climate change) remains a worthy goal. After decades of research, the key ingredients have been identified, and the goal is now in sight. Although it will take more time to come to fruition, it seems certain to happen. It will not be realized as quickly as it could be, or should be, however, because many practitioners are still pursuing SM couplings that rely on 1–5 mol% of EAM-based catalysts (or less) in organic solvents, implying environmental negligence. This is most unfortunate because these SM couplings being worked on by both academic and industrial laboratories are consuming precious resources, especially in the fine chemical industry, which has better alternatives at their disposal, right now. There is no long-term future in organic solvents, especially when Nature has proven over geological time that water is its chosen medium. Sooner or later, water will be the accepted “solvent” for a “perfect” Group 10 metal-catalyzed SM couplings and related reactions, whether in a “in water” or an “on water” sense. It is hoped that when the “perfect” conditions for a SM coupling are found, it will not be too late.

## Data Availability

No publicly available datasets were analyzed in this study.
